# ‘Above all, do no harm’: safeguarding pluripotent stem cell therapy against iatrogenic tumorigenesis

**DOI:** 10.1186/scrt462

**Published:** 2014-06-03

**Authors:** Marek Malecki

**Affiliations:** 1 Phoenix Biomolecular Engineering Foundation, San Francisco, CA 94105-9992, USA; 2 NMRFM, National Institutes of Health, Madison, WI 53706-1526, USA; 3 University of Wisconsin, Madison, WI 53706-1526, USA

## Abstract

Human pluripotent stem cells are the foundations of regenerative medicine. However, the worst possible complication of using pluripotent stem cells in therapy could be iatrogenic cancerogenesis. Nevertheless, despite the rapid progress in the development of new techniques for induction of pluripotency and for directed differentiation, risks of cancerogenic transformation of therapeutically implanted pluripotent stem cells still persist. 'Above all, do no harm', as quoted from the Hippocratic Oath, is our ultimate creed. Therefore, the primary goal in designing any therapeutic regimes involving stem cells should be the elimination of any possibilities of their neoplasmic transformation. I review here the basic strategies that have been designed to attain this goal: sorting out undifferentiated, pluripotent stem cells with antibodies targeting surface-displayed biomarkers; sorting in differentiating cells, which express recombinant proteins as reporters; killing undifferentiated stem cells with toxic antibodies or antibody-guided toxins; eliminating undifferentiated stem cells with cytotoxic drugs; making potentially tumorigenic stem cells sensitive to pro-drugs by transformation with suicide-inducing genes; eradication of differentiation-refractive stem cells by self-triggered transgenic expression of human recombinant DNases. Every pluripotent undifferentiated stem cell poses a risk of neoplasmic transformation. Therefore, the aforementioned or other novel strategies that would safeguard against iatrogenic transformation of these stem cells should be considered for incorporation into every stem cell therapy trial.

## Introduction

Healthy cells of an embryo developing *in utero* are pluripotent. From a single cell zygote, they proliferate into the trillions of cells in an adult human. They also differentiate, through the three main germ layers, into the adult cells of tissues and organs. Most of the mature cells do not proliferate, but fulfill complex physiological processes, for example, neurons or cardiomyocytes. Some of the cells keep proliferating and differentiating as part of their regular *modus operandi* (for example, bone marrow or skin epithelium). During development, these two processes are accompanied by a third, apoptosis - selective cell death of *non gratae* cells in maturing organs. This balance is destroyed in various processes of pathology, of which at least two are worth discussing here.

**Table 1:** Strategies safeguarding pluripotent stem cell therapy against iatrogenic cancerogenesis

**Authors**	**Stem cells**	**Key reagent**	**Procedure**	**Mechanism**
Fong *et al.*[28]	Human embryonic	Mouse monoclonal antibodies anti-SSEA-4, anti-TRA-1-60	FACS, MACS	Depletion of SSEA-4+, TRA-1-60 + hESCs
Tang *et al.*[30]	Human embryonic	Mouse monoclonal antibodies anti-SSEA-5, CD9, CD90 or anti-SSEA-5, CD50, CD200	FACS	Depletion of SSEA-5+ hESCs
	Human pluripotent induced			
Ben-David *et al.*[31]	Human embryonic	Mouse monoclonal antibody anti-claudin-6	FACS	Depletion of claudin-6+ hESCs
				Killing by toxins linked to antibodies
	Human induced pluripotent			
King *et al.*[29]	Human embryonic	Mouse monoclonal antibodies anti-SSEA-4, anti-TRA-1-60, anti-FGFR-1, anti-CD133, anti-CD13519	FACS	Separation of fate-specific subpopulations of GFP + hESCs
Malecki [11]	Human autologous induced pluripotent	Synthetic antibodies anti-SSEA-4, anti-TRA-1-60	MACS, FACS	Depletion of SSEA-4+, TRA-1-60+ non-differentiating hiPSCs
Gerrard *et al.*[32]	Human embryonic	Fusion protein expressed from *OCT4-EGFP*	Transfection with lentivirus vector	Monitoring and selection of differentiating cells
Eiges *et al.*[33]	Human embryonic	Fusion protein expressed from *Rex1 (murine promoter)-EGFP*	Electroporation LipofectAMINE, FuGENE, ExGen500	Monitoring and selection of undifferentiated cells
Nishimori *et al.*[16]	Murine induced pluripotent	Fusion protein expressed from *NANOG-GFP*	Selection in embryoid bodies	Discarding undifferentiated cells from EBs
van Laake *et al.*[34]	Murine induced pluripotent	Fusion protein expressed from *NKX2-5-EGFP*	FACS	Selection of differentiating cells
Chung S *et al.*[35]	Murine embryonic	Fusion protein expressed from *SOX1-GFP*	FACS	Selection of differentiating cells
Malecki *et al.*[10]	Human autologous induced pluripotent	Fusion fluorescent proteins expressed under promoters for *OCLN5*, *CLDN5*, *TJP1*, *PECAM1*, *CDH5*, *CTNNB*	Transfection with synthetic antibody-guided vector	Monitoring and selecting of differentiating cells
Choo *et al.*[36]	Human embryonic	Mouse monoclonal antibody anti-PODXL	Antibody dissolved in media	Killing undifferentiated cells
Schriebl *et al.*[37]	Human embryonic	Mouse monoclonal antibody anti-PODXL	Antibody dissolved in media	Sorting of undifferentiated cells + killing with toxic antibody
Lim *et al.*[38]	Human embryonic	ScFv 84-HTH antibody fragment anti- PODXL	Antibody dissolved in media	Killing undifferentiated cells in EBs prior to transplantation
Ben-David *et al.*[39]	Human induced pluripotent	PluriSIn#1	Reagent dissolved in media	Inhibition of SCD1 leading to UPR, ER stress, and apoptosis
Conesa *et al.*[40]	Murine embryonic	Benzethonium chloride, methylbenzethonium chloride	Reagent dissolved in media	Mitochondrial membrane permeability, apoptosis
	Human induced pluripotent			
Lee *et al.*[41]	Human embryonic Human induced pluripotent	Quercetin, YM155	Reagent dissolved in media	Mitochondria-mediated selective cell death
Vazquez-Martin *et al.*[42]	Murine induced pluripotent	Metformin	Reagent dissolved in media	Apoptosis
Bieberich *et al.*[43]	Human embryonic Human induced pluripotent	S18 (N-oleoyl serinol)	Reagent dissolved in media	Apoptosis
Schuldiner *et al.*[44]	Human embryonic	HSV-TK under *PGK* promoter + GCV	Transfection with plasmid in ExGen500	Apoptosis
Hara *et al.*[45]	Human embryonic	HSV-TK under *OCT4* promoter + GCV	Transfection with viral vector	Apoptosis
Rong *et al.*[46]	Human embryonic	HSV-TK gene into *NANOG* locus + GCV	Homologous recombination	Apoptosis
Cheng *et al.*[47]	Human embryonic	HSV-δTK under mouse *NANOG* or *EF1α*promoter + GCV	Transfection with lentiviral vector	Apoptosis
	Human induced pluripotent			
Chen *et al.*[48]	Murine undifferentiated pluripotent stem	Recombinant cytosine deaminase under mouse *NANOG* or *EF1α*promoter + 5-fluorocytosine	Transfection with lentiviral vector	Apoptosis
Malecki *et al.*[50]	Human autologous induced pluripotent	Human recombinant *DNASE1*, *DNASE1L3*, *DNASE2*, *DFFB* under *POLA1* promoter	Transfection with anti-SSEA-4-guided vector	Apoptosis + necrosis

*EB*, embryoid body; *EGFP*, enhanced green fluorescent protein; *ER*, endoplasmic reticulum; *FACS*, fluorescence activated cell sorting; *FGFR*, fibroblast growth factor receptor; *GCV*, ganciclovir; *GFP*, green fluorescent protein; *hESC*, human embryonic stem cell; *hiPSC*, human induced pluripotent stem cell; *HSV*, herpes simplex virus; *MACS*, magnetic activated cell sorting; *SCD1*, stearoyl-CoA desaturase 1; *SSEA*, stage specific embryonic antigen; *TK*, thymidine kinase; *TRA*, tumor related antigen; *UPR*, unfolded protein response.

First, in cancer, cells acquire the ability to proliferate in an uncontrolled manner. In embryonal carcinomas, the cells have many features identical to the cells of developing embryos. In anaplastic cancers, the cells dedifferentiate and lose the features of the tissues from which they originated, so that the lineage of their origin is nearly impossible to determine. In teratomas, the cells proliferate and differentiate, but not in synchrony with the entire body and its established architecture. Apoptotic and repair mechanisms should ensure that cancer cells are eliminated and tissues reconstructed, but instead these mechanisms are either mutated, disabled, or cannot discriminate cancer cells from healthy cells. Thus, discovering the mechanisms of proliferation, differentiation, immune response, and death may lead not only to cures for cancers, but also therapies that guide the processes of regeneration.

Second, in cases of tissue injury, the body initiates rescue and regeneration responses. Adult cells gain the ability to replace damaged cells and to heal the injured tissue completely and with fully restored functions, but without triggering tumor formation (for example, epithelium in skin injuries). Unfortunately, in some cases the natural responses are not sufficient (for example, cardiac stem cells in myocardial infarctions). The means to control these responses and to enhance them using pharmacotherapeutics or transgenes are not yet within our clinical management repertoire. Administration of pluripotent cells is aimed at supporting the regeneration processes of injured tissues, but without inflicting iatrogenic harm.

Stem cells form the therapeutic basis for regeneration of organs injured by disease, for reconstruction of tissues damaged by iatrogenic effects of therapies, for rejuvenation of systems affected by aging, for correction of congenital defects caused by genetic mutations, and even for delivery of therapeutic transgenes [1-5]. This wide spectrum of potential applications is based upon the unique capabilities of pluripotent stem cells not only to self-renew into the same kind of undifferentiated cells, but also to differentiate into a variety of specialized tissues. Biomarkers displayed on the surfaces of undifferentiated cells, and that change upon differentiation of these cells, are the same for human embryonal carcinomas of ovaries and testes, human embryonic stem cells (hESCs), and human induced pluripotent stem cells (hiPSCs). Importantly, induced pluripotent stem cells (iPSCs) do not present ethical concerns associated with destroying human embryos needed for gaining embryonic stem cells [6,7]. Moreover, autologous human induced pluripotent stem cells (ahiPSCs) reduce the risks of rejection of implanted cells by the immune system of recipients [8]. The essential problem for successful regenerative therapy, recruitment and retention of pluripotent stem cells to the sites of therapeutic intervention, has recently been addressed by introduction of genetically engineered, heterospecific, polyvalent antibodies [9-11]. However, all engineered pluripotent stem cells carry a risk of neoplastic transformation, which has prompted work on measures to safeguard against this [12-19]. In fact, the first reported induced stem cells were generated by transfection with sets of genes for transcription factors known as strong oncogenes (*MYC* and *KLF4*). Furthermore, they were cloned into integrating, and thus potentially cancerogenic, retroviral vectors. Accordingly, one of the tests for pluripotency of these cells is their ability to form teratomas in nude mice. Current research is moving towards using non-integrating viral vectors, changing the number and composition of transcription factors, enhancing and simplifying induction of pluripotency by small molecules, the use of systems that ensure differentiation of progenitors into the desired lineage, as well as towards bypassing induction of pluripotency altogether by directed trans-differentiation [20-27].

Nevertheless, despite rapid progress in the development of new methods for inducing pluripotency and for directed differentiation, completely erasing genomic memory may require solid reversal of differentiation, and differentiation refractive cells may contaminate batches of therapeutic cells planned for therapeutic implantation. Indeed, several cases of neoplasms grown from implanted iPSCs have been reported. Therefore, the serious risk of neoplasmic transformation of induced pluripotent stem cells persists.

## Review of safeguarding strategies

'Above all, do no harm', as quoted from the Hippocratic Oath, is our ultimate creed. Therefore, the primary goal in designing any therapeutic regime involving stem cells should be the elimination of any possibilities for their neoplastic transformation. I review here the basic strategies that have been designed to attain this goal: sorting out undifferentiated, pluripotent stem cells with antibodies targeting surface-displayed biomarkers; sorting in differentiating cells, which express recombinant proteins as reporters; killing undifferentiated stem cells with toxic antibodies or antibody-guided toxins; eliminating undifferentiated stem cells with cytotoxic drugs; making potentially tumorigenic stem cells sensitive to pro-drugs by transformation with suicide-inducing genes; and eradication of differentiation-refractive stem cells by self-triggered transgenic expression of human recombinant DNases.

### Sorting out undifferentiated, pluripotent stem cells

Undifferentiated, pluripotent stem cells display unique surface biomarkers. Stage specific embryonal antigen SSEA-3 and SSEA4 are the most established biomarkers of pluripotent stem cells displayed on embryonic stem cells (ESCs), iPSCs, and cells of embryonal carcinomas of the testes and ovaries. Display of these biomarkers is quickly downregulated upon differentiation of the stem cells. However, stem cells refractive to differentiation retain their surface display profiles. Monoclonal antibodies raised in mice or nano-antibodies and aptamers synthesized chemically target these stem cells’ biomarkers. Each of them, after being modified with fluorochromes or superparamagnetic chelates, may facilitate fluorescence activated cell sorting (FACS) or magnetic activated cell sorting of the undifferentiated, pluripotent stem cells.

Antibodies against SSEA-3 and SSEA-4 and tumor related antigen 1–60 and 1–81 (TRA-1-60 and TRA-1-81) were first developed and used for not only monitoring, but also selection of undifferentiated ESCs and iPSCs. For example, after directing differentiation of ahiPSCs towards the endothelial or myocardial lineages, the undifferentiated cells were sorted out by superparamagnetic or fluorescent antibodies [9-11,28,29]. This procedure eliminated potentially tumorigenic stem cells.

SSEA-5 was identified on human pluripotent stem cells. Monoclonal antibody raised against SSEA-5 facilitated separation of SSEA-5+ and SSEA-5- cell populations [30]. After implanting an under the kidney capsule in immunodeficient mice, all SSEA5+ cells grew into tumors (7 out of 7 tested), while SSEA-5- cells reduced the number of tumors (3 out of 11 tested). As such, the antibodies to SSEA-5 were insufficient alone to separate all residual tumorigenic cells. Therefore, further reduction of sizes and numbers of forming tumors was accomplished by combining antibodies against SSEA-5 with two other antibodies, resulting in two combinations: SSEA-5, CD9, and CD90; or SSEA-5, CD50, and CD200 [30].

Claudin-6 is a member of the claudin family, which contributes to formation of tight junctions. It is displayed on hESCs and hiPSCs but is dispensable for their survival and renewal [31]. This molecule is also expressed on cancer cells, influencing their anchorage properties. It is retained during pluripotency. Fluorescently modified monoclonal anti-claudin-6 antibodies facilitated separation by FACS of cells displaying claudin 6 from those not displaying it. After injection of these two populations into immunodeficient mice, tumors grew from only the claudin-6-positive cells, but not from the claudin-6-negative cells. This strategy was further enhanced by linking the anti-claudin-6 antibody with toxins (see below).

Mouse monoclonal antibodies anti-SSEA-4, anti-TRA-1-60, anti-fibroblast growth factor receptor 1, anti-CD133, and anti-CD135 were used to separate subpopulations of stem cells from green fluorescent protein (GFP) + hESCs. GFP+/CD133+ cells differentiated toward ectoderm as determined by gene expression for nestin, which was not the case with GFP+/CD133- cells. CD135+/GFP + and CD135- hESCs gave rise to tissues representing all three embryonic germ layers. These studies suggest lineage preferences within hESCs rather than uniform pluripotency. In such cases, these subpopulations may differ in their response to differentiating factors, as well as in their tumorigenicity [29].

### Sorting in differentiating cells expressing green fluorescent protein as reporters

Inducing and maintaining pluripotency involves expression of specific genes encoding the appropriate pluripotency transcription factors. The most critical are *NANOG*, *OCT4*, and *SOX2*. Directed differentiation of iPSCs involves monitoring the expression of differentiation lineage-specific genes. For example, expression of *MEF* and *GATA* is the earliest sign of myocardial differentiation. Transgenic expression of *OCLN5* (endothelial occludin), *CLDN5* (endothelial claudin), *PECAM1* (platelet/endothelial cell adhesion molecule 1), *CDH5* (cadherin), *TJP1* (zona occludens), and *CTNNB* (catenin) is the earliest sign of vasculogenesis [10]*.* Genes for GFPs and their mutants, under control of promoters or fused with coding sequences for the proteins, which are uniquely specific for undifferentiated or differentiated cells, serve well as reporters of these phenomena. If undifferentiated pluripotent stem cells are transformed to express GFPs as reporters, then as long as they remain undifferentiated, they emit fluorescence upon illumination with specific wavelengths. This facilitates their sorting out with FACS or eradication through laser ablation. If differentiating cells in cultures or embryoid bodies are expressers, then only differentiating cells can be selected and non-fluorescent cells can be abandoned. Both approaches have been applied to monitor fates of ESCs and iPSCs.

The hESCs were transfected with constructs for *EGFP* (enhanced GFP) driven by the *OCT4* promoter (*OCT4-GFP*) within lentiviral vectors [32]. In tests for embryoid body formation and *in vitro* differentiation, their expression was validated as representing endogenous expression of *OCT4* in undifferentiated hESCs. *OCT4* small interfering RNA downregulated this expression, resulting in reduced differentiation [32]. Similar studies were conducted by expressing *EGFP* under the *Rex1* (*REX-EGFP*) murine promoter [33]. In both cases, fluorescent reporters facilitated identification and separation.

hiPSCs were transfected with GFP constructs under the *NANOG* promoter. This resulted in expression of the fluorescent transcription factor (*NANOG-GFP*) in undifferentiated cells. Upon formation of embryoid bodies, expression of GFP indicated the presence of undifferentiated cells. GFP-positive and -negative embryoid bodies were injected into nude mice; the first developed tumors, while the latter did not. This approach guided selection of non-tumorigenic cells within embryoid bodies.

An alternative strategy was developed by genetic engineering constructs coding for fluorescent proteins under control of genes expressed in differentiating cells or as fusions with those genes’ products. Expression of fluorescent reporters served as a guide for selection of progenitors for further differentiation. Mouse iPSCs were transfected with the transgene for a fusion protein of the early cardiac transcription factor NKX2-5 and a GFP reporter (NKX2-5-GFP) and then directed to differentiate [34]. Fluorescent transgene expression products facilitated selection of NKX2-5/GFP + cardiac progenitors, while undifferentiated cells were discarded. Similarly, mouse ESCs were transfected to express GFP under the *SOX1* promoter (SOX1-GFP) [35]. This provided an opportunity for positive selection of fluorescing cells.

ahiPSCs were directed to differentiate into endothelial cells as a means to regenerate infarcted myocardium by revascularization. The processes of differentiation were monitored by combinations of transduction with vectors carrying genetically engineered constructs for GFP and its mutants as fusions with OCLN5 (endothelial occludin), CLDN5 (endothelial claudin), PECAM1 (platelet/endothelial cell adhesion molecule 1), CDH5 (cadherin), TJP1 (zona occludens), and CTNNB (catenin) and labeling with superparamagnetic and fluorescent synthetic antibodies against those proteins [10].

### Killing pluripotent stem cells with cytotoxic antibodies or antibody-guided toxins

Immunotherapy for cancer relies upon direct cytotoxicity or the ability to elicit immune cytotoxic responses by selected clones of antibodies towards cancer cells with surface-displayed mutated and/or upregulated gene expression products - cell surface displayed cancer biomarkers [31]. Alternatively, the antibodies can be linked to toxins, chemotherapeutics, cancer cell suicide-inducing genes, or radionuclides, which then serve as carriers delivering deadly cargo to the targeted cancer cells. Ideal examples of such strategies are provided by therapies built around *EGFRvIII*, which encodes a mutant epidermal growth factor receptor missing a large portion of its extracellular domain. As a result, a portion of this extracellular domain has a different molecular structure to the corresponding portion in the wild-type receptor and is thus a different immunogen. This creates the difference in immunogenic specificity and absence of cross-reactivity between the antibodies against those receptors. Therefore, EGFRvIII is a unique target for developing targeted therapies, vaccines, and immunotherapeutics. The same principles are applied to developing strategies that utilize antibodies to destroy undifferentiated stem cells. First and foremost, however, these strategies rely upon identification of specific biomarkers - molecules displayed exclusively on surfaces of undifferentiated, pluripotent stem cells.

Podocalyxin like protein 1 has been identified on the surfaces of stem cells in hESC lines (HES-2, HES-3, HES-4) and in a pluripotent cell line (NCCIT) derived from non-seminomatous germ cell tumors. A monoclonal antibody against this protein demonstrated cytoxicity against undifferentiated cells, but did not interfere with the progress of differentiating cells [36-38]. Moreover, since natural antibodies with their high molecular weights (IgG, 150 kDa; IgM, 750 kDa) do not penetrate well into embryoid bodies and tissues, single chain variable fragments were engineered with a much smaller molecular weight (20 kDa) and a thus much smaller hydrodynamic cross-section. This resulted in much better penetration and much higher efficacy in elimination of potentially tumorigenic stem cells [38].

Claudin-6 is one of the biomarkers of undifferentiated pluripotent hESCs and hiPSCs. Monoclonal antibodies against claudin-6 have been modified with fluorochromes and used for sorting out these cells by FACS in order to reduce the risk of tumor growth. As an alternative strategy, this antibody was used to guide toxins to the marked stem cells. After attaching to the stem cells, this mouse monoclonal antibody was a target for an anti-mouse monoclonal antibody delivering a saporin toxin [31]. Upon this targeted delivery, the toxin had a selective, devastating effect on the claudin-6-expressing stem cells.

### Treatment with cytotoxic molecules

Progression of cancer is driven by uncontrolled proliferation of cancer cells. Chemotherapies exert cytostatic and/or cytotoxic effects on these proliferating cancer cells. The clinical efficacy of chemotherapies is contingent upon their selective discrimination, in intake and efficacy, between cancer and healthy cells. The greater the intake by cancer cells and/or the greater the sensitivity of cancer cells to a drug relative to healthy cells, then the better the therapeutic efficacy and the lower the amount of side effects. Therefore, therapeutic doses in clinical oncology are at levels that maximize killing of cancer cells (effective doses) while minimizing harm to healthy cells (side effects). The same principle is proposed when using drugs for killing pluripotent stem cells threatening to develop into neoplasms.

PluriSIn#1 demonstrated selective efficacy in elimination of undifferentiated iPSCs and ESCs [39]. To identify this drug, human pluripotent stem cells in matrigel were exposed for 12 hours to various compounds from a high-throughput screen of 52,448 small molecules - a subset of the Hoffmann-La Roche diverse chemical entities compound library. PluriSIn#1 inhibits stearoyl-CoA desaturase-1, an enzyme involved in metabolism of monounsaturated fatty acid. This leads to apoptosis of the treated cells [38].

Benzethonium chloride and methylbenzethonium chloride are quaternary ammonium salts. They are better known as effective antimicrobial agents, including for treatment of the 'superbug' methicillin-resistant *Staphylococcus aureus*, which killed more people than AIDS in 2005. They have also been used as anticancer agents. They were selected after screening a library of 1,120 chemicals to identify those that induce death of undifferentiated ESCs [40]. Their pharmacological mechanism of action involves loss of mitochondrial membrane potentials and activation of caspases. This leads to triggering of the apoptotic signaling pathways and activation of the DNases. They were effective in killing murine ESCs, while having no effect on murine adult fibroblasts. Similarly, human fibroblast-derived induced stem cells were sensitive to these salts, but sourced fibroblasts were not.

Quercetin and YM155 were used to treat undifferentiated hESCs, iPSCs, and spontaneously differentiated cells from embryoid bodies [41]. Both reagents did not affect processes of differentiation, which were tested by relative expression levels of lineage-specific differentiation marker genes (*AFP*, *FOXA2*, *GATA6* for endoderm; *Brachyury T*, *TnTc*, *IGF2* for mesoderm; and *PAX6*, *NCAM* for ectoderm). These molecules inhibited formation of tumors in nude mice after xenografting [40].

Metformin suppressed *Oct4* in stem cells without interfering with the Oct4-independent abilities of these cells to differentiate into tissues [41]. This is an additional application of a drug regularly used for treatment of diabetes. As the outcome, it prevented occurrence or caused reduction in the size and weight of teratoma-like masses after transplantation of the murine iPSCs into immunodeficient mice [42].

In tumors formed after engraftment of embryoid body cells into mouse brains, Oct4 colocalized with prostate apoptosis response-4 (PAR-4), a protein mediating ceramide-induced apoptosis during neural differentiation of hESCs. Treatment of undifferentiated stem cells with the ceramide analogue N-oleoyl serinol (S18) induced formation of a complex between PAR-4 and protein kinase C zeta, which resulted in inhibition of PAR-4 and apoptosis of the treated cells. Untreated cells formed numerous tumors [43].

*Clostridium perfringens* enterotoxin binds specifically to members of the claudin protein family. Upon binding of this toxin by a cell, the cell's membrane permeability rapidly changes, resulting in the cell's death. Since pluripotent, undifferentiated stem cells display claudin-6 they are a target for this toxin. Indeed, 1 hour *in vitro* treatment of undifferentiated stem cells in culture was sufficient to kill all of them [31]. *In vivo*, no tumors were detected in mice treated with *Clostridium perfringens* enterotoxin (none out of four), while all the untreated, xenografted mice developed tumors (four out of four) [31].

### Killing pluripotent stem cells by transfection with genes inducing cell suicide upon exposure to pro-drugs

Herpes simplex virus (HSV)-thymidine kinase (TK) in combination with ganciclovir (GCV) is the most widely used strategy for suicide gene therapy of cancer [4,44-48]. Unlike cellular TK, HSV-TK has a range of specificities, including pyrimidine, the pyrimidine analog deoxycytidine, guanosine, and the acyclic guanosine nucleoside analogue GCV. Although GCV is the pro-drug of choice, its application is limited due to high toxicity. In non-transfected cells, cellular TK catalyzes the transfer of phosphate from ATP to thymidine to produce dMTP. In transfected cells, HSV-TK catalyzes this reaction also for the mentioned analogs. These nucleoside analogs, once phosphorylated, are further phosphorylated by cancer cell kinases to triphosphates. Some of these analogs, including GCV, are capable of inhibiting DNA synthesis through arrest at the G2/M check point, which triggers apoptosis. Transportation of the phosphorylated analogs through the gap junction or via apoptotic vesicles to the neighboring cells leads to their apoptosis. This is known as a bystander effect. This strategy has been successfully adapted to kill undifferentiated stem cells.

The plasmid construct was engineered to contain the gene for HSV-TK under the *PGK* promoter (*PGK-HSV-TK*) [44]. It was transfected using ExGen500 into hESCs. Upon exposure to varying concentrations of GCV (2 × 10^-8^ M to 2 × 10^-5^ M) *in vitro*, the transfected hESCs died, while the non-transfected hESCs were unaffected. The efficacy was quantified based upon flow cytometry of hESCs stably transformed with GFP. Some of the transfected cells reversed (1 × 10^-6^ to 1 × 10^-7^). The hESCs were also xenografted into immunodeficient mice. Tumor growth was inhibited after treatment with GCV, but progressed in the absence of such treatment in control mice.

*PGK-HSV-TK* presented a risk of affecting all proliferating cells. To reduce that risk, a viral vector was engineered in which HSV-TK was set under the control of the *OCT4* promoter [45]. After transforming hESCs, this strategy limited the effects of GCV treatment to undifferentiated cells only with active transcription factors unique to the pluripotent stem cells [45].

The viral vector may promote cancerogenesis through random incorporation that disrupts cancer suppressing or repairing genes. To address this problem, the bacterial artificial chromosome vector carrying *NANOG* was re-engineered by inserting an IRES-TKSR39-IRES-Puro-IRES-EGFP expression cassette flanked by two *LoxP* sites. This *HSVTK* construct was inserted into the *NANOG* locus by homologous recombination [46]. After xenografting into nude mice, tumor formation was eliminated in mice treated with GCV (0/10), compared with high tumorigenicity in non-treated mice (4/4). Furthermore, the *HSV-TK* transduced cells were effectively directed to differentiate with retinoic acid, while tested for *PAX6* (ectoderm), *PECAM* (mesoderm), *3SCL*, and *AFP* (endoderm) gene expression. Therefore, the construct did not interfere with these cells' capability to differentiate.

Suicide gene transfection was also applied to reduce the risk of iatrogenic cancerogenesis in hiPSCs. The hESCs and hiPSCs were infected with lentivirus engineered to deliver *HSV-δTK* under control of the *NANOG* or *EF1* promoters [47]. The genetically modified cells retained their ability to self-renew and differentiate. *In vitro*, almost no *HSV-δTK* modified cells were still viable in the group treated with 5 μg/ml for 5 days, while there were no toxic effects on the non-modified cells. The rate of apoptosis increased to 90% on the fifth day of treatment. *In vivo*, the most significant result of the study was selective ablation of undifferentiated pluripotent stem cells and progression of differentiating cells towards the three germ layers.

Cytosine deaminase catalyzes hydrolysis of cytosine to uracil with release of ammonia. If endonucleases recognize modified sites, then the phosphodiester bonds are broken, while initiating repair by incorporation of new cytosines. This pharmacological mechanism is utilized for cancer suicide gene therapy - that is, if the non-toxic pro-drug 5-fluorocytosine is provided, then cytosine deaminase converts it into 5-fluorouracil, which inhibits cancer cell growth. This strategy has been successfully applied in selective killing of tumor-initiating murine pluripotent stem cells by expressing recombinant cytosine deaminase or δTK under control of the *EF1α* or *NANOG* promoters [48].

### Eradication of human induced pluripotent stem cells by proliferation-triggered transgenic expression of recombinant DNases

Despite great advances towards development of reliable strategies for eliminating potentially tumorigenic cells from batches planned for therapeutic implantation, a couple of issues are worth improving before translating these strategies into clinics. First, the discussed strategies do not have a self-triggered safeguarding mechanism, but instead require constant monitoring, which is not easy in patients. Second, they require preventive application of pro-drugs, but we know from oncology clinics that all the pro-drugs have serious side effects, in particular GCV. Third, pro-drugs may cause mutations in genomic DNA in the germ cells, introducing the risk of genetic disorders in offspring. These reasons have prompted work on developing a strategy that would incorporate a self-triggered feed-back loop while not relying upon provision of toxic reagents. Protecting the fertility of patients suffering from cancers and eliminating the risk of genetic disorders in their offspring present identical problems, and attained solutions are considered here.

Cancer development is driven by uncontrolled proliferation of cancer cells. The key element of their proliferation involves passing the G1 > S check-point, which initiates replication of genomic DNA. In various cancers, elements of this transition are strongly stimulated by complex and multiple signaling pathways. For example, in brain, lung, testicular, or ovarian cancers, expression of the *EGFR* gene, which encodes the epidermal growth factor receptor (EGFR), is greatly upregulated. Targeted therapies aim to interrupt these pathways, but cancer cells have the ability to develop alternative pathways, to repair damaged DNA, to expel therapeutics, and to reverse apoptotic processes, as long as they retain intact genomic DNA. Considering the aforementioned data, an entirely different strategy has been developed, consisting of genetically engineered DNA constructs for the recombinant DNases controlled by the *EGFR* promoter, synthetic antibody-guided biotag-targeted delivery of these constructs only into cancer cells expressing mutated EGFRs or over-expressing them, expression only in cancer cells, and intranuclear trafficking of the transgenically expressed DNases [49].

The same principles guided development of the strategy aimed at eradication of all undifferentiated pluripotent stem cells [50]. The ahiPSCs were directed to differentiate into endothelial or myocardial lineages. Thereafter, these cells were transfected with vectors carrying the transgenes for human recombinant *DNASE1*, *DNASE1L3*, *DNASE2*, and *DFFB*, while guided by anti-SSEA-4 and anti-TRA-1-60 synthetic antibodies. Therefore, the vectors delivered the transgenes to pluripotent, differentiation-refractive stem cells. Myocardial differentiation was monitored by expression of GFP and its mutants controlled by *MEF* and *GATA* promoters. Early endothelial differentiation was monitored by expression of *GFP* controlled by *OCLN5*, *CLDN5*, *PECAM1*, *CTNNB*, or *CDH5* promoters. Proliferation was monitored by expression of GFP mutants controlled by the *POLA1* promoter. If the hiPSCs did not differentiate but retained their pluripotency and specific cell surface display profiles and kept proliferating, then they expressed the human recombinant DNases, which were completely effective in degrading genomic DNA, thus causing the death of these potentially tumorigenic stem cells.

## Future directions

The first sets of transcription factors used to reprogram adult cells into iPSCs relied on provision of potent oncogenes (*MYC* and *KLF*) and transcription factors known to be overexpressed in undifferentiated stem cells. Since then, several other combinations of factors inducing pluripotency have been designed and tested, but a lot of work remains to be done [17-20]. The immediate studies will define the complete spectra of pluripotency-inducing transcription factors and the multiple pathways/mechanisms controlling them, so that full pluripotency can be attained and controlled differentiation can be pursued without a risk of tumorigenesis *in vivo*. The results of these studies will affect two strategies of clinical practice. First, autologous pluripotent stem cells will be generated by dedifferentiation of donors’ adult cells, expanded and induced to differentiate into therapeutically desired lineages. After identification of the exact patterns of gene expression during development, it may not be necessary to fully erase the epigenetic memory of adult cells, but only to the stage sufficient for the lineage switch. Moreover, it may become possible to alter their immunological profiles, so that they will not trigger rejection if used as heterologous transplants. Second, identification of these genes’ expression patterns will open opportunities for direct lineage reprogramming without dedifferentiation to the pluripotency stage at all [10,21]. Seamless, functional integration with the host’s healthy tissue will be the ultimate test of whether any new approaches are successful.

Not only function, but also integration of regenerating cells into the architecture and the correct location in the host’s organs is essential. Recruitment and retention of the iPSCs in sites of therapeutic intervention have been significantly enhanced by anchoring them with the heterospecific polyvalent antibodies. Aforementioned advances in controlling the processes of differentiation will direct further pursuits of that strategy in clinical trials. First, hiPSCs will be anchored through the displayed receptors [9-11]. This approach will require application of all of the safeguards against tumorigenesis reviewed above. Second, as an alternative, the progenitors generated through *in vitro*-induced differentiation or through directed trans-differentiation will display lineage-specific biomarkers that will serve as tagging points for anchoring them with heterospecific antibodies. Thorough sorting in of progenitors and sorting out of undifferentiated cells will eliminate the risk of tumorigenesis.

Current reprogramming technologies have mostly been pursued *in vitro*, but recently progressed into trials *in vivo* as extensively reviewed [26]. In this realm, enhancement of patients’ natural regenerative potential will rely upon targeted delivery of pharmacotherapeutics or transgenes *in vivo* aimed at triggering expression of genes promoting healing, while suppressing expression of genes promoting tumorigenesis. This will present the next level of challenges for attaining desired efficacy in targeting, reprogramming, and differentiation.

The first and foremost step in the preparation of stem cells for therapeutic implantation requires that they have to be thoroughly characterized and carefully sorted with high throughput and under good manufacturing process regimes to assure selection of large quantities of the most homogeneous populations featuring high viability, the required characteristics, and desired potential. This effort is aided by identification of specific biomarkers displayed on the surface of stem cells and the lineage-specific progenitors, and developing antibodies or aptamers against these biomarkers, and modifications that make them suitable for isolation on sorters. SSEA-1, SSEA-3, and SSEA-4 were initially the targets for developing mouse monoclonal antibodies. However, they also became the targets for developing synthetic nano-antibodies and aptamers with superparamagnetic or fluorescent properties to make them suitable for high speed magnetic or fluorescent sorters [9-11]. Research towards identification of new biomarkers and development of new antibodies or aptamers will surely be vigorously pursued. As an alternative, incorporation of recombinant reporters under pluripotency, differentiation, or inducible promoters will continue. GFPs and their mutants under the control of *OCT4* or *NANOG* promoters are good examples of such approaches [10,11,32-34,50]. With progress in research on stem cell genomes, transcriptomes, and proteomes, new genes of interest will surely be identified and other reporter systems designed.

Finally, one of the most pressing issues to address in relation to all stem cell-based therapies is monitoring the fate of the administered cells *in vivo*. This will be pursued by continued refinement of the already developed strategies for tracking their localization upon and after administration, monitoring processes of differentiation towards the desired lineages, detection of tumorigenesis, or reporting deaths of therapeutically delivered cells.

## Conclusion

Therapeutic stem cells pose a potential risk of neoplastic transformation. Therefore, the strategies reviewed here, or novel strategies, that would safeguard against iatrogenic transformation of these stem cells should be considered for incorporation into every stem cell therapy trial.

## Abbreviations

ahiPSC: autologous human induced pluripotent stem cell; EGFP: Enhanced green fluorescent protein; EGFR: Epidermal growth factor receptor; ESC: Embryonic stem cell; FACS: Fluorescence activating cell sorting; GCV: Ganciclovir; GFP: Green fluorescent protein; hESC: human embryonic stem cell; hiPSC: human induced pluripotent stem cell; HSV: Herpes simplex virus; Ig: Immunoglobulin; iPSC: induced pluripotent stem cell; PAR-4: Prostate apoptosis response-4; SSEA: Stage specific embryonic antigen; TK: Thymidine kinase; TRA: Tumor related antigen.

## Competing interests

MM holds the rights to the intellectual property for the gene constructs and their transcripts and products used in the cited works, which are all protected at USPTO and WIPO.
